# Long acting risperidone in Australian patients with chronic schizophrenia: 24-month data from the e-STAR database

**DOI:** 10.1186/1471-244X-12-25

**Published:** 2012-03-26

**Authors:** Tim Lambert, Brett Emmerson, Harry Hustig, Sophie Resseler, An Jacobs, Belinda Butcher

**Affiliations:** 1 Department of Psychiatry, The University of Melbourne, Melbourne, Australia; 2 Concord Medical School and Brain and Mind Research Institute, University of Sydney, Camperdown, NSW, Australia; 3 Mental Health Services, Royal Brisbane and Women's Hospital, Herston, QLD, Australia; 4 Extended Care Services, Royal Adelaide Hospital, North Terrace Adelaide, SA, Australia; 5 Life Science Services--Biometrics, SGS, Antwerp, Belgium; 6 Health Economics & Pricing, Janssen Pharmaceutica, Beerse, Belgium; 7 Medical Research, Janssen-Cilag Pty Ltd, North Ryde, NSW, Australia; 8 WriteSource Medical Pty Ltd, Lane Cove, NSW, Australia

## Abstract

**Background:**

This observational study was designed to collect treatment outcomes data in patients using the electronic Schizophrenia Treatment Adherence Registry (e-STAR).

**Methods:**

Patients with schizophrenia or schizoaffective disorder in Australia who were prescribed risperidone long-acting injection (RLAI) between 2003 and 2007 were assessed 12-months retrospectively, at baseline and 24-months prospectively at 3-monthly intervals. The intent-to-treat population, defined as all patients who received at least one dose of RLAI at baseline, was used for the efficacy and safety analyses.

**Results:**

At total of 784 patients (74% with schizophrenia, 69.8% male) with a mean age of 37.1 ± 12.5 years and 10.6 ± 9.5 years since diagnosis were included in this Australian cohort. A significant improvement in mean Clinical Global Impression - severity score was observed at 24-months (4.52 ± 1.04 at baseline, 3.56 ± 1.10 at 24-months). Most of this improvement was seen by 3-months and was also reflected in mean Global Assessment of Functioning score, which improved significantly at 24-months (42.9 ± 14.5 at baseline, 59 ± 15.4 at 24-months). For patients still receiving RLAI at 24-months there was an increase from a mean baseline RLAI dose of 26.4 ± 5 mg to 43.4 ± 15.7 mg. Sixty-six percent of patients discontinued RLAI before the 24-month period--this decreased to 46% once patients lost to follow-up were excluded.

**Conclusion:**

Over the 24-month period, initiation of RLAI was associated with improved patient functioning and illness severity in patients with schizophrenia or schizoaffective disorder. Improved outcomes were observed early and sustained throughout the study.

**Trial Registration:**

Clinical Trials Registration Number, NCT00283517.

## Background

Approximately 800,000 hospital patient care days per year are associated with treatment of schizophrenia or schizoaffective disorders [1], which represents a significant cost to the Australian healthcare system. There is increasing evidence that a lack of adherence to prescribed antipsychotic medication is a predictor for relapse and subsequent hospitalisation [2,3]. Adherence is increased by between 10 and 40% with the use of long-acting injectable antipsychotics [4]. However, there is concern regarding "the excessive use of [conventional] depot medication as a crude response to the widespread lack of adherence to oral medications" [5], given the increased risk of tardive dyskinesia and higher levels of extrapyramidal symptoms associated with these agents compared to atypical antipsychotics [5]. Risperidone long-acting injection (RLAI), the first long-acting formulation of an atypical antipsychotic, offers an alternative to conventional depot medication.

However, to date, little information is available on the treatment, practice and outcomes potential of RLAI outside of the clinical trial setting. In 2002, the lack of information outside of clinical trials led the National Institute for Health and Clinical Excellence (NICE) in the United Kingdom to recommend more high quality observational studies be performed to assess key questions around the effects of atypical agents [6]. It was upon this basis that the electronic Schizophrenia Treatment Adherence Registry (e-STAR) database was developed to collect treatment outcomes of patients prescribed RLAI for schizophrenia or schizoaffective disorder.

The objectives of eSTAR were to: prospectively assess medication usage patterns, to document clinical efficacy and long-term treatment outcomes of RLAI, in a naturalistic setting; to collect retrospective data, which allow the evaluation of treatment outcomes with RLAI compared to previous treatments, and to evaluate reasons for initiating RLAI.

## Methods

The e-STAR is a multi-country, observational study that collects treatment outcomes data in patients with schizophrenia using web-based electronic data-capture. The primary objectives of the study were to evaluate drug use patterns, efficacy and long-term treatment outcomes of long-acting risperidone injection in a naturalistic setting. Data collection was accomplished via a secure web-based system. In order for the registry to capture real-life treatment information, there were no formalised diagnostic procedures, randomisation or treatment decisions mandated in the protocol. Physicians based their treatment decisions on their best medical judgment. Data for the Spanish cohort have been published previously [7]. The present paper describes results from the e-STAR study in Australia, which was collected between October 2003 and March 2007 at 15 clinical sites across Australia. The study was approved by the relevant institutional ethics committee at each centre, was conducted in compliance with the Declaration of Helsinki, and registered: Clinical Trials Registration Number, NCT00283517. Ethics approval numbers for each of the centres can be found in the Ethics Approval section at the end of this manuscript.

### Participants

Patients with schizophrenia or schizoaffective disorder (diagnoses on the basis of DSM-IV criteria [8]) who started treatment with RLAI at an in- or out-patient setting during the course of their routine clinical management were eligible to participate. Patients with co-morbid psychiatric disorders were eligible to participate. All participants provided written, informed consent prior to any data collection, either personally, or via their appointed legal guardian.

### Antipsychotic therapy

Participants were prescribed RLAI according to the Australian local label [9]. For risperidone naïve patients, it was recommended that a test dose of immediate release oral formulation was given to establish tolerability [9]. The recommended starting dose for all patients was 25 mg given intramuscularly every two weeks [9]. Sufficient antipsychotic therapy coverage was recommended during the three-week lag period following the first RLAI injection [9]. For patients switched from a long-acting depot medication, RLAI was initiated at the next scheduled depot injection time, with sufficient oral coverage required for 3-weeks. Upward dose adjustments were recommended to not be made more frequently than every four weeks [9].

### Data collection

Data collection in the study has been described previously [7]. Briefly, however, three data collection periods were specified: retrospective, baseline and prospective. The retrospective data were collected from patient medical records from the treating hospital and/or community mental health clinic for a period of 12-months prior to initiation of RLAI. Baseline data were collected at the time of initiation of RLAI. Prospective data were collected every 3-months from baseline for a period of 24-months.

Data collected during the retrospective period included treatment and hospitalisation history. At baseline (defined as the initiation of RLAI) the following information was collected: patient demographics, illness characteristics (using the Clinical Global Impression of illness Severity [CGI-S [10]] and Global Assessment of Functioning [GAF [8]]) including dose of RLAI, the reason for RLAI initiation, other psychiatric medications, and community treatment orders (CTO). Prospectively, we collected information on CGI-S, GAF, psychiatric hospitalisations, concomitant therapies, clinical deterioration (defined as one of the following: partial or full hospitalisation for exacerbation of psychotic symptoms; need to increase level of care combined with an increase in CGI-S of at least 2 points; the emergence of suicidal or homicidal ideation; deliberate self harm, and violent behaviours), treatment adherence and discontinuation, compulsory treatment orders, and adverse events.

### Statistical analyses

Data were entered directly into the web-based e-STAR tool. Data presented here are based upon intent-to-treat (ITT) and per protocol (PP) analyses of Australian patients enrolled in this 24-month study. The ITT population were those subjects who received at least one injection of RLAI. The PP population were those subjects who received RLAI at baseline and completed the study (defined as having at least one recorded piece of information 720 days (24-months) post baseline). The PP population did not necessarily remain on RLAI for the 24-month period. We did not use a last observation carried forward procedure. Therefore, comparisons between the PP and ITT populations can only be made at baseline. Safety data are presented for the ITT and PP populations of Australian patients.

Descriptive statistics are reported for patient demographics, disease characteristics, treatment adherence, treatment discontinuation, reasons for RLAI initiation, RLAI dose, antipsychotic and other concomitant medications, and adverse events. Differences in the number of hospitalisations were analysed using the signed-rank test, while differences in length of hospitalisation were compared using paired t-tests. Changes in medications and clinical deterioration were calculated under the *a priori *hypothesis of no change from baseline to 24-months using the McNemar test. Changes in CGI-S and GAF were compared using paired t-tests.

A Kaplan-Meier procedure was used to estimate the percentage of patients who discontinued from RLAI in the first 24-months following initiation. The first reason for RLAI discontinuation was reported with descriptive statistics.

Data are presented as mean (standard deviation) unless otherwise noted. To adjust for multiple testing, statistical significance was assigned where P < 0.001.

## Results

### Patient demographics

A total of 784 patients were available for inclusion in the ITT analysis. The PP population consists of 359 patients. The demographic, baseline clinical characteristics and previous hospitalisations of ITT and PP patients are shown in Table 1. For those patients in the ITT population with "other" diagnoses, the diagnoses included schizophreniform (n = 13); delusional disorder (n = 6); bipolar affective disorder (n = 4); psychosis (n = 4); "likely" schizophrenia (n = 1); and schizoaffective with organic personality disorder (n = 1). The ITT and PP were similar with respect to baseline demographics, although those who completed the study (PP population) had a longer duration of illness compared to the whole (ITT) population.

**Table 1:** Patient demographics and baseline clinical characteristics

Characteristic	ITT n = 784	PP n = 359
Age (years, mean ± SD)	37.1 ± 12.5;	39.3 ± 13
(range)	18 to 26	not reported

Sex, n (%)		
Male	547 (69.8%)	246 (68.5%)
Female	237 (30.2%)	113 (31.5%)

Diagnosis		
Schizophrenia	580 (74.0%)	266 (74.1%)
Schizoaffective disorder	175 (22.3%)	84 (23.4%)
Other	29 (3.7%)	9 (2.5%)

Time since diagnosis (years, mean ± SD)	10.6 ± 9.5	12.2 ± 10.4

Unemployed, n (%)	705 (89.9%)	319 (88.9%)

CGI-S score (mean ± SD)	4.52 ± 1.04	4.48 ± 1.04

Mean GAF score (mean ± SD)	42.9 ± 14.5	42.3 ± 14.5

Community treatment order	394 (50.3%)	188 (52.3%)

Full hospitalisations in previous 12-months	604 (77.0%)	267 (74.4%)

*CGI-S *clinical global impression--severity; *GAF *global assessment of functioning

The main reasons patients were initiated on RLAI were due to adherence (51.8%), unacceptable tolerability/adverse events (24.7%), and insufficient response (14.3%). In the 3-months prior to initiation of RLAI, most patients who were on antipsychotic medications received oral atypical antipsychotics either as monotherapy (27.0%) or in combination (13.4%); or were administered a conventional depot medication either as monotherapy (23.1%), or in combination (11.2%).

### Treatment

**Figure 1:**
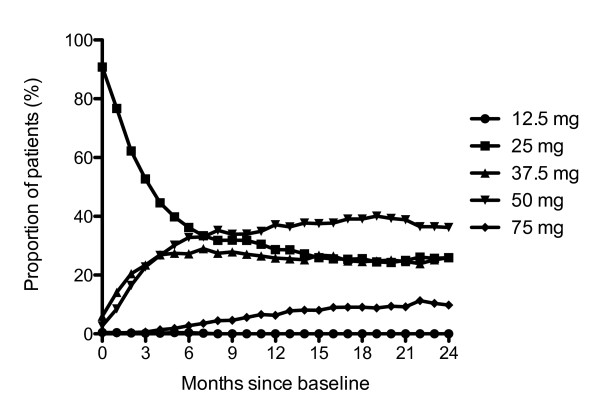
**Proportion of patients on each dose of RLAI over time (N = 784 at baseline)**. The proportion of patients on 12.5 mg was 0.5% at 1 month, 0.4% at 2, 6 and 7 months, 0.3% at 3 months, 0.2% at 4, 5 and 8 months. All other time points were 0%. RLAI, risperidone long-acting injection.

The majority of patients in the ITT population were initiated on 25 mg RLAI (90.8%). The mean dose at baseline was 26.4 ± 5.0 mg with a median of 25 mg. At 24 months, the mean dose in patients still receiving RLAI was 43.4 ± 15.7 mg with a median of 37.5 mg. Slightly more than a third of patients had no dose adjustments (38.0%); others subsequently went on to have 1 (22.3%) or 2 (23.6%) dose adjustments. Three or more dose adjustments were required in only 16.1% of patients. The proportion of patients taking each dose of RLAI over the 24-months is presented in Figure 1.

Similar to the ITT population, the majority of patients in the PP population were initiated on 25 mg RLAI (89.4%). The mean dose at baseline was 26.6 ± 5.2 mg, which increased to 43.6 ± 15.7 mg at 24 months. A greater proportion of patients required 2 dose adjustments (28.4%), than none (22.3%), 1 (25.3%), or 3 or more (24.0%).

### Other psychiatric medications

For PP patients with 24 months of follow up, there was a decrease in the use of other psychiatric medications over the course of the study (70.3% at baseline vs. 64.3% at 24-months, p = 0.048). There was a reduction in benzodiazepine use (42.3% at baseline vs. 29.7% at 24-months, p < 0.001), a non-significant increase in the use of antidepressants (16.9% at baseline vs. 18% at 24-months, p = 0.71), and a reduction in concurrent anticholinergics (22% at baseline vs. 16% at 24-months, p = 0.026). There was no marked change in the use of mood stabilisers or somatic medications. As the percentage of patients at baseline using other psychiatric medications was similar between the ITT (71.0%) and PP (70.3%) populations, the PP population is considered a representative subpopulation of the ITT.

### Illness characteristics

**Figure 2:**
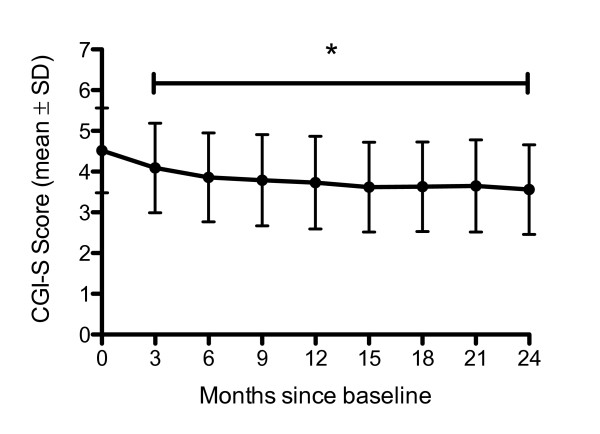
**CGI-S Score over time - all patients**. *Time points were significantly different from baseline (p < 0.001). CGI-S, clinical global impression--severity.

**Figure 3:**
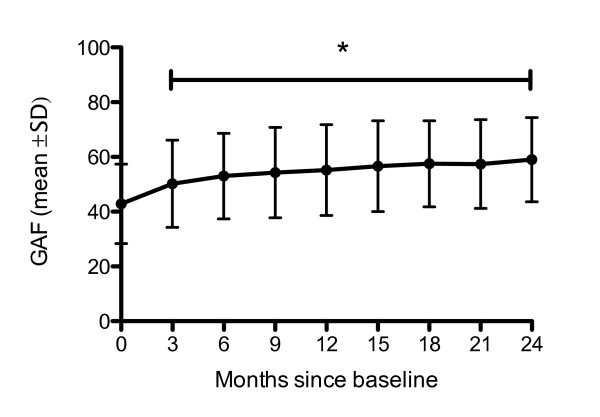
**GAF Score over time--all patients**. *Time points were significantly different from baseline (p < 0.001). GAF, global assessment of functioning.

A significant reduction in mean CGI-S score occurred over the 24-month period (from 4.48 ± 1.04 at baseline to 3.59 ± 1.09 at 24-months; p < 0.001; Figure 2). The majority of this reduction was seen in the first 3-months and sustained over the 24-month period. This pattern was reflected in the GAF scores, which improved significantly over the 24-month period (from 42.3 ± 14.5 at baseline to 58.7 ± 15.3 at 24-months; p < 0.001). Again, the majority of change occurred within the first 3-months and was sustained over the full 24-month period (Figure 3). Similarly, the ITT results were: 4.52 ± 1.04 at baseline to 3.56 ± 1.10 at 24-months. And, were also reflected in the improvement in GAF scores over 24-months: 42.9 ± 14.5 at baseline to 59 ± 15.4 at 24-months.

### Hospitalisations

At baseline, 51.1% of patients were being treated on an in-patient basis. During the 12-months post baseline (using discharge date as the baseline for inpatients), 48.5% of patients were fully hospitalised at least once, as opposed to 77% in the 12-months prior to baseline (p < 0.001). The percentage of patients fully hospitalised at least once post baseline increased from 48.5% at 12-months to 58.1% at 24-months. However, if the initiation date of RLAI is used as a baseline for all patients, 70.2% of patients were fully hospitalised at least once in the first 12-months (including the initial episode). And, by 24-months, the percentage of patients fully hospitalised increased slightly to 73.5%. Only patients with data at the specified time point were included in the analyses (i.e., completers only).

### Clinical deterioration

There was a significant reduction in the proportion of patient rehospitalisations from baseline to 24-months (ITT, 54.2% at baseline vs. 12.7% at 24-months; PP, 47.9% at baseline vs. 12.7% at 24-months, p < 0.001 McNemar's test). There was also a significant reduction in the proportion of patients who were suicidal or exhibited homicidal ideation (ITT: 7.9% at baseline vs. 2.3% at 24-months; PP, 7.6% at baseline vs. 2.3% at 24-months, p = 0.001). Although improved at 24-months compared with baseline, violent behaviour (p = 0.84) and self-injury (p = 0.18) did not show a significant change over the same time period.

### Discontinuation of treatment

**Figure 4:**
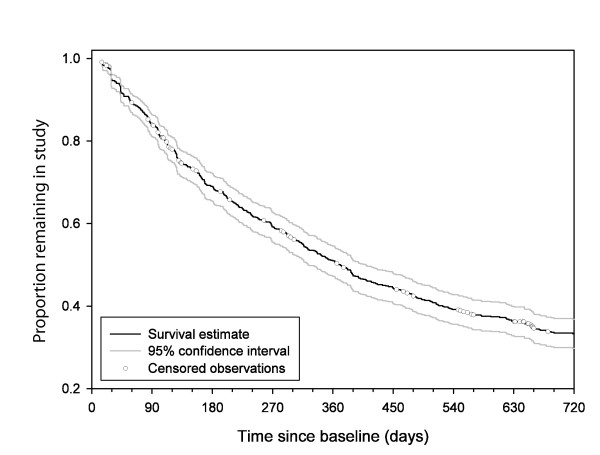
**Time to discontinuation of RLAI (n = 784)**.

The Kaplan-Meier estimate for the percentage of ITT patients who discontinued RLAI before 24-months was 66.7% (95% CI: 63.2% to 70.2%; Figure 4). For the PP population, the Kaplan-Meier estimate was 46.0% (95% CI 41.0% to 51.3%). For the 472 patients in the ITT analysis who discontinued RLAI before 24-months the mean time to discontinuation was 239 ± 180 days. The main reasons cited for discontinuation were insufficient response to treatment (25.8%) and lost to follow-up (23.7%). Excluding patients with lost to follow-up given as the reason for RLAI discontinuation (i.e. patients with no 24-month follow-up); the rate decreases to 46%. The most frequently prescribed antipsychotic at the time of discontinuation of RLAI was oral risperidone (21.6%) either alone (15.3%) or in combination with other antipsychotics (6.3%). Other agents frequently prescribed (either alone or in combination) were olanzapine (9.5%), clozapine (9.1%) and zuclopenthixol (8.1%).

### Adverse events

There were 2273 adverse events reported by 411 (52.4%) patients in the ITT population during the prospective 24-month period of the study. Relationship to RLAI was not reported. The most common events (occurring in > 5% patients) were sedation (10.3%), increased weight (8.7%), headache (7.1%), and tremor (6.5%). Sixty-seven (8.6%) patients reported 153 serious adverse events. The most common (occurring in > 1% of patients) were suicidal ideation (1.4%), death (1.3%), and hospitalisation (1.0%).

Of the 359 patients in the PP population, 194 (54.0%) received RLAI for at least 24-months. A total of 628 events were reported by 109 (56.2%) patients who were still receiving RLAI at study end. The most common events (occurring in > 5% patients) were increased weight (12.4%), sedation and headache (8.2%), tremor (7.7%), akathisia (7.7%), insomnia (5.2%), dyspnoea (5.2%) and vomiting (5.2%). Nineteen (9.8%) patients who received RLAI at study end reported 44 serious adverse events. The most common (occurring in ≥ 1% of patients) were hospitalisation (1.5%), suicidal ideation (1.5%) pneumonia (1.0%), intentional overdose (1.0%), overdose (1.0%), aggression (1.0%) and suicide attempt (1.0%).

## Discussion

The patients recruited into the e-STAR study are representative of the general population of patients with schizophrenia or schizoaffective disorder in Australia, although we possibly included a higher proportion of patients with schizoaffective disorder (22% cf 15% in previous reports [7,11]). However, they were similar in age, sex and duration of illness to those reported in the SOHO study [12]. They were also similar in age, sex, duration of illness and disease severity to those reported in the Spanish report of the e-STAR study [7]. One of the difficulties of research in this area is that a proportion of eligible participants refuse to provide informed consent. As we did not keep a screening log, we are unable to determine what proportion of eligible participants chose not to participate.

One of the important findings of this study is its support of recent findings by Viner and colleagues [13] in that many patients with chronic schizophrenia or schizoaffective disorder may benefit from an up-titration from 25 mg to 37.5 or 50 mg. Viner and colleagues [13] suggest that although 25 mg is frequently the initial RLAI dose many patients require at least 37.5 mg, if not 50 mg, for symptom improvement. To achieve maximal clinical efficacy we found the most common number of titrations was 2 with an increasing proportion of patients on 37.5 and 50 mg dosages during the time-course of the study.

Patients receiving RLAI demonstrated improvements in CGI-S over the duration of the study - with a change in mean category from 4 - 'moderately ill' to 3 - 'mildly ill'. This correlates with a change in BPRS score from 40-45 to 32-36, or a reduction of approximately 10 points [14], indicating a clinically significant change. Considering that reductions in CGI-S scores for more severely ill patients are potentially more important, this finding is clinically notable. This sub-group of ill patients are likely to experience a greater number of symptoms, and reductions are likely to correlate with more substantial symptom improvement, especially given the possibility of ceiling effects [14]. The improvement observed is similar to previously reported reductions in CGI-S scores at 3-months of between 0.5 and 0.9 following initiation of RLAI in patients with schizophrenia [15], and is consistent with the findings of the Spanish e-STAR investigators who reported a reduction in CGI-S score of 1.14 points [7].

Recently, the GAF scale has been validated on the domains of psychological, social and occupational functioning in patients with schizophrenia [16]. We observed improvements in GAF scores of approximately 15 points - a significant improvement in patient functioning. This is consistent with the findings of the Spanish e-STAR investigators who reported an increase in GAF score of 17.3 points [7].

The mechanisms responsible for improvements in disease severity and functioning were not assessed in our study, however, we hypothesize that improvements were due to improved adherence with antipsychotic medication, as has been reported by others [17-19].

Although initiation of RLAI resulted in an overall decrease in the percentage of patients using other psychiatric medications, the changes in concomitant medication use reported in this study are complicated by the collection technique we used to gather these data (a simple tick box of whether the medication had been used in the previous 3-months). As such, we are unable to separate medications taken during an acute admission, such as benzodiazepines, from those that were prescribed on a long-term basis. We know that patients are prescribed benzodiazepines as an alternative to neuroleptic treatment in acute psychosis [20]. Lambert and colleagues [20] report that benzodiazepines are prescribed for 17% of patients prior to admission, peaking at 42% on the first day of admission and reducing to 28% at the time of discharge.

Initiation of RLAI significantly reduced the number of hospitalisations for patients who remained on treatment for 12 and 24 months. This again reflects the findings of the Spanish e-STAR investigators [7]. This finding has potential implications for health care costs in the long-term management of schizophrenia. Additionally, it suggests there may be considerable cost savings over a 24-month period through reduced hospitalisation [21].

The treatment discontinuation rate in this study is high compared to that observed in the RLAI Spanish cohort of the e-STAR study at 24-months: 66.7% vs18.2% [7]. It is not clear why there is this difference in discontinuation between the two countries: baseline demographics were similar in the two cohorts. It may reflect different mental health policies between the two countries, or the itinerant nature of the Australian population. The seemingly poor RLAI retention rate observed in this study, although improved for patients who completed the full 24-month period, may be explained by the population being more treatment resistant, as shown by the choice of post-RLAI antipsychotic treatment. Oral risperidone, olanzapine, clozapine, and zuclopenthixol, alone or in combination, were the most frequently prescribed medications post-RLAI. Furthermore, it is well known patients participating in antipsychotic treatment studies tend to have high dropout rates [22,23]. There was little difference between baseline characteristics of those who completed the study compared to the overall study population (with the exception that the duration of diagnosis was longer in the completer population). Clozapine, the treatment of choice for treatment-resistant patients, was frequently prescribed post-discontinuation of RLAI (9.1%). However, the proportion of patients receiving combinations of antipsychotic therapies suggests that some were resistant to treatment, and therefore likely to be non-adherent.

The adverse event profile was as to be expected in this patient population [9,15,24].

A prominent limitation of this study is the lack of a control arm. The data in this paper are from Australia only, and differences in culture and health care systems may mean our results are not generalisable to other countries. However, our results do support those reported by the Spanish e-STAR group [7]. That said, the population observed is consistent with that seen in Australian practice and is therefore relevant to clinicians in Australia. Furthermore, it should be emphasised that this study was naturalistic (observational), containing both retrospective and prospective elements, however, as it was not blinded, there is the possibility of observational bias.

## Conclusion

The data generated from the e-STAR Australian cohort shows RLAI treatment is associated with early improvements in illness severity and patient functioning, and these improvements are sustained at 24-months. RLAI treatment was also associated with a decrease in hospitalisation rates and in the use of concomitant psychotropic medication.

### Ethics approvals

The following ethics committees provided ethical approval for the study: Alfred Hospital, The Alfred Ethics Committee (48/04); Box Hill Hospital, Eastern Health Research & Ethics Committee (E31/0304); Dandenong Hospital, Southern Health-Human Research Ethics Committee (03139D); Fremantle Hospital, Human Research Ethics Committee-Fremantle Hospital and Health Service (03/324); Glenside Campus, Royal Adelaide Hospital-Research Ethics Committee (030904a); Gold Coast Hospital, Gold Coast Health Service District-Queensland Health (200401); Graylands Hospital, Human Research Ethics Committee, Area Mental Health Services, North Metropolitan Health Service (1.15.13 (03/15)); Hornsby Ku-ring-gai Hospital, Northern Sydney Health-Human Research Ethics Committee (0311-220 M); Royal Brisbane Hospital, Royal Brisbane & Women's Hospital & Health Service District Office of the Human Research Ethics Committee (2003/157); Royal Hobart Hospital, Department of Health and Human Services-Tasmania, (H0007646); Royal Melbourne Hospital, Melbourne Health Behavioural and Psychiatric Research & Ethics Committee (E/04/004); St Vincent's Hospital, St Vincent's Health, Research and Grants Unit (HREC-D 139/03); The Prince Charles Hospital, Human Research Ethics Committee, The Prince Charles Hospital Health Service District (EC2366); The Queen Elizabeth Hospital, Central Northern Adelaide Health Service-Ethics of Human Research Committee (2003116); and Westmead Hospital, Western Sydney Area Health Service-Human Research Ethics Committee (HREC2003/12/3.2(1712)).

## Abbreviations

RLAI: Risperidone long-acting injection; CGI-S: Clinical global impression of illness severity; GAF: Global assessment of functioning; ITT: Intent-to-treat; PP: Per protocol.

## Competing interests

Tim Lambert, Harry Hustig and Brett Emmerson are consultants, supported speakers and supported investigators of Janssen-Cilag Pty Ltd, Australia. At the time the study was conducted, An Jacobs and Belinda Butcher were employees of Janssen Pharmaceutica and Janssen-Cilag Pty Ltd, respectively. These companies market Risperdal CONSTA. Sophie Resseler, an independent statistician, was contracted to perform biostatistical services by Janssen Pharmaceutica. Janssen-Cilag Pty Ltd provided funding for publication of the manuscript.

## Authors' contributions

TL, BE, and HH contributed to the acquisition of data, revising the drafted manuscript for important intellectual content and provided final approval of the version to be published. SR analysed the data, revised the drafted manuscript for important intellectual content and provided final approval of the version to be published. AJ contributed to the conception and design of the study, revised the drafted manuscript for important intellectual content and provided final approval of the version to be published. BB contributed to the quality control in acquisition of data, interpretation of the data, drafting the manuscript and provided final approval of the version to be published. All authors read and approved the final manuscript.

## Pre-publication history

The pre-publication history for this paper can be accessed here:

http://www.biomedcentral.com/1471-244X/12/25/prepub
